# Genome-wide methylation profiling identifies signatures of pain, fatigue and health scores in women with systemic lupus erythematosus

**DOI:** 10.1093/rheumatology/keag392

**Published:** 2026-07-30

**Authors:** Alessandro Camponeschi, Amin Ravaei, Tahzeeb Fatima, Chris Wincup, Anna Rudin, Jan Bjersing, Cristina Maglio

**Affiliations:** Department of Rheumatology and Inflammation Research, Institute of Medicine, Sahlgrenska Academy, University of Gothenburg, Gothenburg, Sweden; Clinical Immunology and Transfusion Medicine, Region Västra Götaland, Sahlgrenska University Hospital, Gothenburg, Sweden; Department of Rheumatology and Inflammation Research, Institute of Medicine, Sahlgrenska Academy, University of Gothenburg, Gothenburg, Sweden; Wallenberg Centre for Molecular and Translational Medicine, University of Gothenburg, Gothenburg, Sweden; Department of Rheumatology and Inflammation Research, Institute of Medicine, Sahlgrenska Academy, University of Gothenburg, Gothenburg, Sweden; Department of Rheumatology, King’s College Hospital, London, UK; Department of Rheumatology and Inflammation Research, Institute of Medicine, Sahlgrenska Academy, University of Gothenburg, Gothenburg, Sweden; Rheumatology Clinics, Sahlgrenska University Hospital, Gothenburg, Sweden; Department of Rheumatology and Inflammation Research, Institute of Medicine, Sahlgrenska Academy, University of Gothenburg, Gothenburg, Sweden; Rheumatology Clinics, Sahlgrenska University Hospital, Gothenburg, Sweden; Department of Rheumatology and Inflammation Research, Institute of Medicine, Sahlgrenska Academy, University of Gothenburg, Gothenburg, Sweden; Wallenberg Centre for Molecular and Translational Medicine, University of Gothenburg, Gothenburg, Sweden; Rheumatology Clinics, Sahlgrenska University Hospital, Gothenburg, Sweden

**Keywords:** SLE, epigenetics, pain, fatigue, women

## Abstract

**Objectives:**

People with systemic lupus erythematosus (SLE) experience high levels of pain and fatigue with poor overall health, which persist in those with low disease activity. By performing epigenome-wide DNA methylation analysis, this study aims to identify epigenetic alterations associated with self-reported scores for pain, fatigue and health in women with SLE.

**Methods:**

Forty-eight women with SLE from the SLEGOT cohort were included. Study participants exhibited low disease activity (median SLEDAI-2K = 0) and minimal damage (median SLICC damage index = 0). An epigenome-wide DNA methylation analysis in whole blood identified 704 237 CpG loci, with 511 673 annotated to known genes.

**Results:**

We identified 485, 591 and 577 differentially methylated CpGs linked to pain, fatigue and poor health, respectively. The association of reported pain with CpGs in *GPR107*, *SPHK2*, *HBA1* and *RERE* genes suggested a potential role for neuromodulation in pain perception in SLE. For fatigue, enrichment analysis highlighted pathways related to neuronal development, morphogenesis and synaptic signalling. Nine genes, including *BDNF* and *TGIF1*, showed strong correlations with all three scores, suggesting a shared epigenetic influence that may underlie pain, fatigue and poor health in SLE. Specific microRNA genes were differentially methylated in relation to pain and fatigue.

**Conclusion:**

By studying a cohort of women with well-controlled SLE, we identified several CpGs and genes associated with pain, fatigue and general health. Our findings suggest that epigenetic changes in genes involved in neuronal modulation, rather than inflammatory pathways, could be involved in the development of these symptoms in patients with SLE.

## Graphical abstract

**Graphical Abstract:**
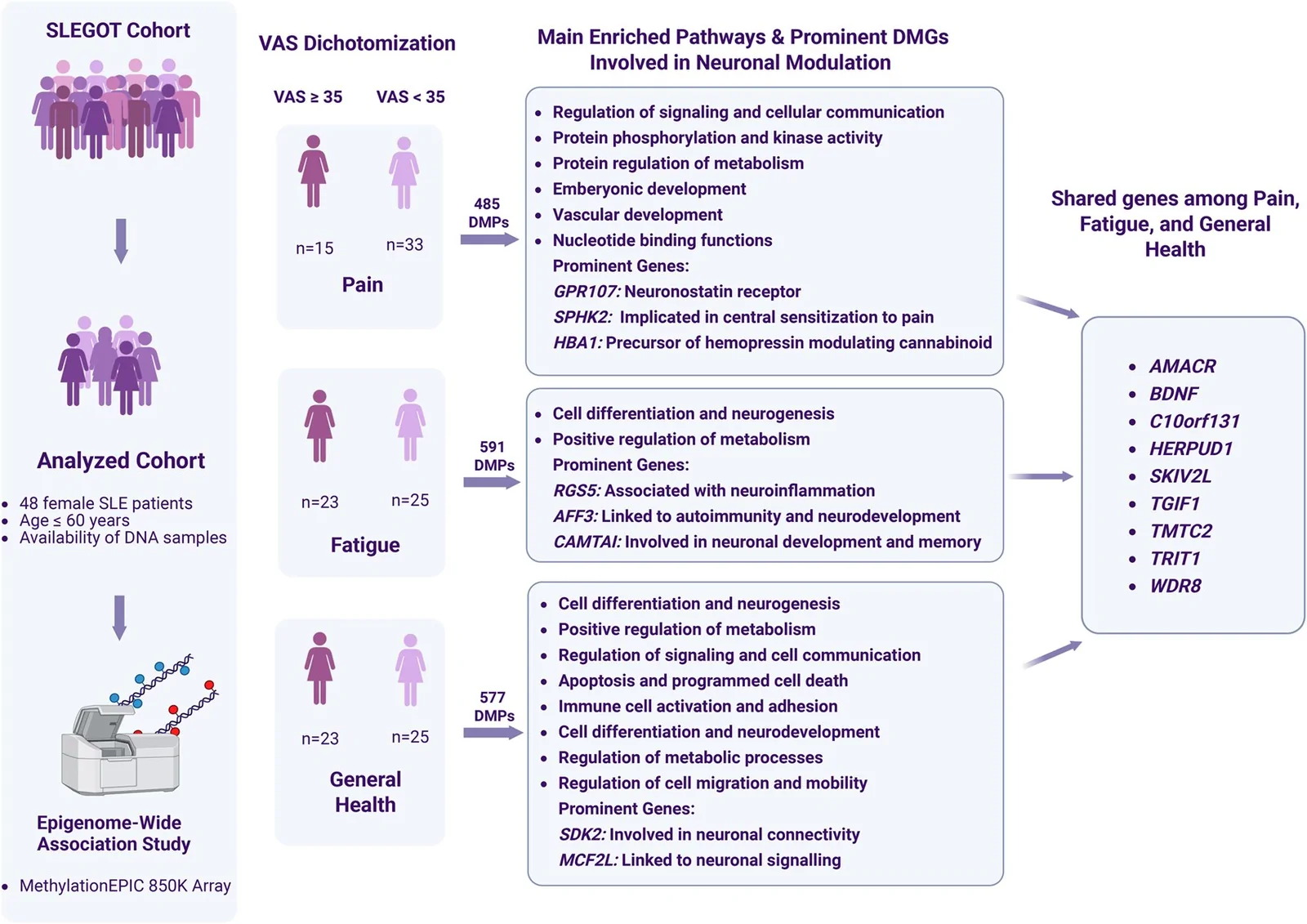

Rheumatology key messages

- Pain, fatigue and poor health persist in SLE despite low disease activity.
- Epigenetic signatures in blood link these symptoms to neuronal pathways rather than inflammation.
- Shared DNA methylation changes suggest common mechanisms underlying pain, fatigue and health in SLE.

## Introduction

Persistent pain and fatigue are frequently observed in patients with systemic lupus erythematosus (SLE) and significantly impact their daily activities, overall well-being and health-related quality of life [1, 2]. Whilst these symptoms can be associated with active disease, they frequently persist in those with low disease activity or remission. The persistence of pain and fatigue in well-controlled patients with SLE is an important unmet need in this group of patients [3, 4].

Self-reported pain, fatigue and overall impaired health in patients with SLE despite clinical remission suggest that there are other factors besides disease activity contributing to the overall burden of the disease, highlighting the need for a deeper understanding of the underlying mechanisms. Pain is an unpleasant sensory and emotional experience related to potential tissue damage, acting as a protective mechanism [5]. In SLE, initial focal tissue damage and inflammation lead to the development of pain [6]. Over time, chronic pain is associated with changes in the nervous system, making the body more sensitive to pain through pathophysiological processes of peripheral and central sensitization. Fatigue is also a major problem among patients with SLE, with up to 90% of patients describing it as their most debilitating symptom [7]. Currently, our knowledge of the underlying mechanisms of chronic pain and fatigue in SLE is very limited [6]. Previous studies across various diseases have shown changes in mRNA and protein expression associated with chronic pain and fatigue, often mediated by underlying epigenetic regulation [8, 9].

Epigenetics is the study of DNA alterations that control gene expression without altering the genome sequence. One important epigenetic modification is the addition of a methyl group to the 5′ position of cytosine in cytosine phosphate guanosine (CpG) pairs, which is mediated by enzymes called DNA methyltransferases. DNA methylation prevents the binding of transcription factors and RNA polymerase, thereby affecting gene expression. Previous studies in SLE show dysregulation of the epigenetic machinery, including effects on DNA methylation and non-coding RNAs, especially microRNAs (miRNAs) [10, 11]. DNA methylation is particularly relevant for studying patient-reported outcomes, as it integrates genetic predisposition with environmental exposures over time. Compared with static genetic variation and the transient nature of gene expression, DNA methylation is relatively stable while remaining responsive to factors such as chronic inflammation, psychological stress and long-term disease burden. These properties make it well suited to capturing biological processes that may underlie persistent symptoms such as pain and fatigue. To determine whether epigenetic alterations correlate with self-reported scores of pain, fatigue and impaired health in patients with SLE with low disease activity, we performed an epigenome-wide DNA methylation study of whole blood in a group of women with long-term well-controlled SLE. We also specifically examined epigenome-wide DNA methylation in miRNA genes.

## Methods

### Study population

From 2017 to 2018, 122 (107 women) SLE patients were recruited at Sahlgrenska University Hospital in Gothenburg, Sweden (SLEGOT Cohort). All participants met the American College of Rheumatology 1997 criteria for SLE diagnosis [12, 13]. This study includes a subset of the SLEGOT cohort, selected based on inclusion criteria of female sex, age ≤60 years, availability of DNA samples and complete data, including clinical symptoms, disease severity, biochemical data and visual analogue scales (VAS) for pain, fatigue, general health and the Health Assessment Questionnaire (HAQ). Self-reported intensity of pain, fatigue and general health was measured on a VAS from 0 to 100. SLE disease activity was assessed using the SLE Disease Activity Index 2000 (SLEDAI-2K, 0–105) [14], and damage was evaluated using the Systemic Lupus International Collaborating Clinics (SLICC) Damage Index [15]. The study protocol was approved by the regional ethics committee of Gothenburg (Dnr 198-17 and T1072-17), and participants provided written informed consent.

### Collection and preparation of samples

Whole blood was collected by venipuncture and was frozen and stored at −80°C. Fifty-six samples were shipped on dry ice to the Bioinformatics and Expression Analysis Core Facility (BEA) Karolinska Institute, Stockholm, Sweden, where genomic DNA was prepared and quality and quantification checked by Qubit Fluorometer (Thermo Fisher Scientific, Waltham, MA, USA). The DNA samples were sodium bisulphite-converted using the Zymo EZ 96 DNA methylation kit (Zymo Research, Irvine, CA, USA; cat no. D5004) according to the manufacturer’s instruction.

### Genome-wide DNA methylation profiling

The methylation levels of converted DNA were analysed using the EPIC 850K Bead-Chip array EPIC 850K Bead-Chip array (Illumina, Inc., San Diego, CA, USA), following the manufacturer’s protocol. The analysis was performed by BEA in collaboration with the Science for Life Laboratory (SciLifeLab; Rudbeck Laboratory, Uppsala University, Uppsala, Sweden). The raw data were subsequently processed using the ChAMP methylation analysis package in R [16] for quality control. During this process, CpG sites that did not pass quality control or were unsuitable due to low specificity were excluded. Furthermore, CpG sites overlapping with single nucleotide polymorphisms having a minor allele frequency >1% were also removed. Subsequently, beta-mixture quantile dilation normalization was applied to obtain the final normalized β-values for DNA methylation levels at the retained CpG sites.

### Statistical analysis

The VAS assessments for pain, fatigue and health were dichotomized to classify participants into two groups based on a threshold score of 35, following previous publication [17]. Participants were categorized into ‘high’ (VAS ≥ 35) and ‘low’ (VAS < 35) groups for each domain. Comparisons between baseline clinical characteristics between ‘high’ and ‘low’ VAS groups were performed using Student’s *t*-test or Wilcoxon’s test for continuous variables and the χ^2^ or Fisher’s exact test for categorical variables, wherever appropriate. Differences in methylation levels between the two groups in each domain were assessed using an independent *t*-test, with adjustment for influential covariates including batch effect, age, ethnicity, BMI, smoking status, disease activity and cell type composition. Statistical significance was first determined using a false discovery rate (FDR) threshold of *q* ≤ 0.05. However, as no CpG sites passed the FDR threshold, we applied a stringent raw *P*-value cutoff of 0.001 for all analyses.

Statistical analysis and data visualizations, including principal component analysis (PCA) and heatmaps, were performed using Qlucore Omics Explorer 3.10.21 (Qlucore AB, Lund, Sweden). Manhattan plots were generated using the ‘qqman’ package in R (https://www.R-project.org/) to provide a genome-wide view of differentially methylated positions (DMPs) across chromosomes, highlighting CpG sites with the most significant associations with pain, fatigue and health scores.

Functional enrichment analysis was conducted using the web-based toolset g:Profiler (https://biit.cs.ut.ee/gprofiler). The Benjamini–Hochberg FDR correction method was applied to account for multiple testing with a significance threshold of ≤0.01. This method adjusts for the number of functional terms tested while considering the structure of the data, such as term sizes and overlaps, to reduce false positives. Terms containing a gene count ≤5000 were included in the analysis. The enriched terms were further analysed and visualized as functional networks using Cytoscape 3.10.3 (http://www.cytoscape.org) and EnrichmentMap (http://www.baderlab.org).

## Results

### Study population

Fifty-six women in the SLEGOT cohort fulfilled the inclusion criteria. Of these, 48 were included in the final analysis, while eight were excluded due to failing DNA methylation quality control (Fig. S1). The clinical characteristics of the analysed cohort (*n* = 48) and the enrolled patients (*n* = 56) are presented in Table 1 and Table S1, respectively. Study participants had a median age of 49 years (interquartile range 33–53) and a disease duration of 16 years (interquartile range 10–26, Table 1). Participants had low levels of established damage (median SLICC damage index 0, interquartile range 0–1) and well controlled disease (median SLEDAI-2K 0, interquartile range 0–1), as well as low levels of CRP and ESR.

**Table 1:** Characteristics of the study cohort (*n* = 48).

Characteristic	All participants (*n* = 48)	Pain			Fatigue			Health		
		High (*n* = 15)	Low (*n* = 33)	*P-*value	High (*n* = 23)	Low (*n* = 25)	*P-*value	High (*n* = 23)	Low (*n* = 25)	*P-*value
Age, years	49 (33–53)	52 (38–57)	47 (33–52)	0.17	49 (33–56)	48 (36–52)	0.90	50 (33–56)	46 (34–52)	0.37
Ethnicity, European, %	90	91	87	0.64	91	88	0.99	96	84	0.35
Disease duration, years	16 (10–26)	20 (11–31)	15 (9–25)	0.34	11 (8–24)	18 (10–27)	0.50	16 (8–25)	15 (10–27)	0.87
Smoking, yes, %	52	45	67	0.29	52	52	0.99	48	56	0.78
Waist circumference, cm^a^	87 (80–101)	89 (11–103)	86 (70–101)	0.48	90 (80–105)	86 (80–100)	0.42	86 (80–103)	87 (81–101)	0.91
BMI, kg/m^2^	24 (22–29)	28 (24–31)	24 (21–28)	0.04	26 (24–31)	24 (21–27)	0.05	25 (22–30)	24 (22–28)	0.58
Systolic BP, mmHg^b^	120 (110–130)	127 (117–139)	120 (110–130)	0.09	120 (115–132)	120 (110–130)	0.18	120 (114–132)	120 (110–130)	0.25
Diastolic BP, mmHg^b^	75 (70–80)	80 (70–80)	73 (70–80)	0.28	77.5 (70–80)	72.5 (70–80)	0.42	78 (72–80)	70 (70–80)	0.12
VAS for pain	12 (6–47)	61 (37–68)	8 (0–12)	<0.001	45 (17–65)	6 (2–9)	<0.001	51 (17–65)	6 (2–9)	<0.001
VAS for fatigue	33 (11–62)	66 (27–72)	22 (0–38)	<0.001	62 (49–71)	11 (5–24)	<0.001	60 (41–71)	11 (5–24)	<0.001
VAS for general health	29 (6–51)	52 (28–64)	9 (1–35)	<0.001	50 (40–62)	6 (4–16)	<0.001	52 (41–64)	6 (4–15)	<0.001
HAQ	0.1 (0.0–0.6)	0.8 (0.5–1.3)	0 (0–0.13)	<0.001	0.5 (0.3–1)	0 (0–0.1)	<0.001	0.5 (0.4–0.8)	0 (0–0.1)	<0.001
EQ5D^c^	0.8 (0.7–1.0)	0.7 (0.6–0.8)	0.9 (0.8–1)	<0.001	0.7 (0.7–0.8)	1 (0.8–1)	<0.001	0.7 (0.7–0.8)	1 (0.8–1)	<0.001
SLICC Damage Index	0 (0–1)	0 (0–1.5)	0 (0–1)	0.95	0 (0–1.5)	0 (0–1)	0.57	0 (0–1)	0 (0–1)	0.42
SLEDAI-2K	0 (0–1)	0 (0–0)	0 (0–1)	0.34	0 (0–2)	0 (0–1)	0.44	0 (0–1.5)	0 (0–1)	0.82
CRP, mg/L	1 (1–2)	3 (1–5.5)	1 (1–4)	0.16	3 (1–6.5)	1 (1–2)	0.01	3 (1–5.5)	1 (1–2)	0.03
ESR, mm/h^d^	14 (5–21)	17 (6–20)	14 (5–26)	0.99	17 (6–24)	11 (5–17)	0.35	16 (5–20)	13 (5–21)	0.99

Values are median (IQR) except where otherwise indicated. There was a total of 48 patients, after exclusion of eight patient samples with a failed CpG fraction above 0.01.

a *n* = 45;

b *n* = 42;

c *n* = 44;

d *n* = 47.

Abbreviations: BP: blood pressure; EQ5D: EuroQol- 5 Dimension score; HAQ: Health Assessment Questionnaire; SLEDAI-2K: SLE Disease Activity Index 2000; SLICC: Systemic Lupus International Collaborating Clinics damage index; VAS: visual analogue scale.

#### DNA methylation profiling

Epigenome-wide DNA methylation analysis identified 704 237 CpG loci that passed quality control. Of these, 511 673 were annotated to known genes.

### Pain

**Figure 1:**
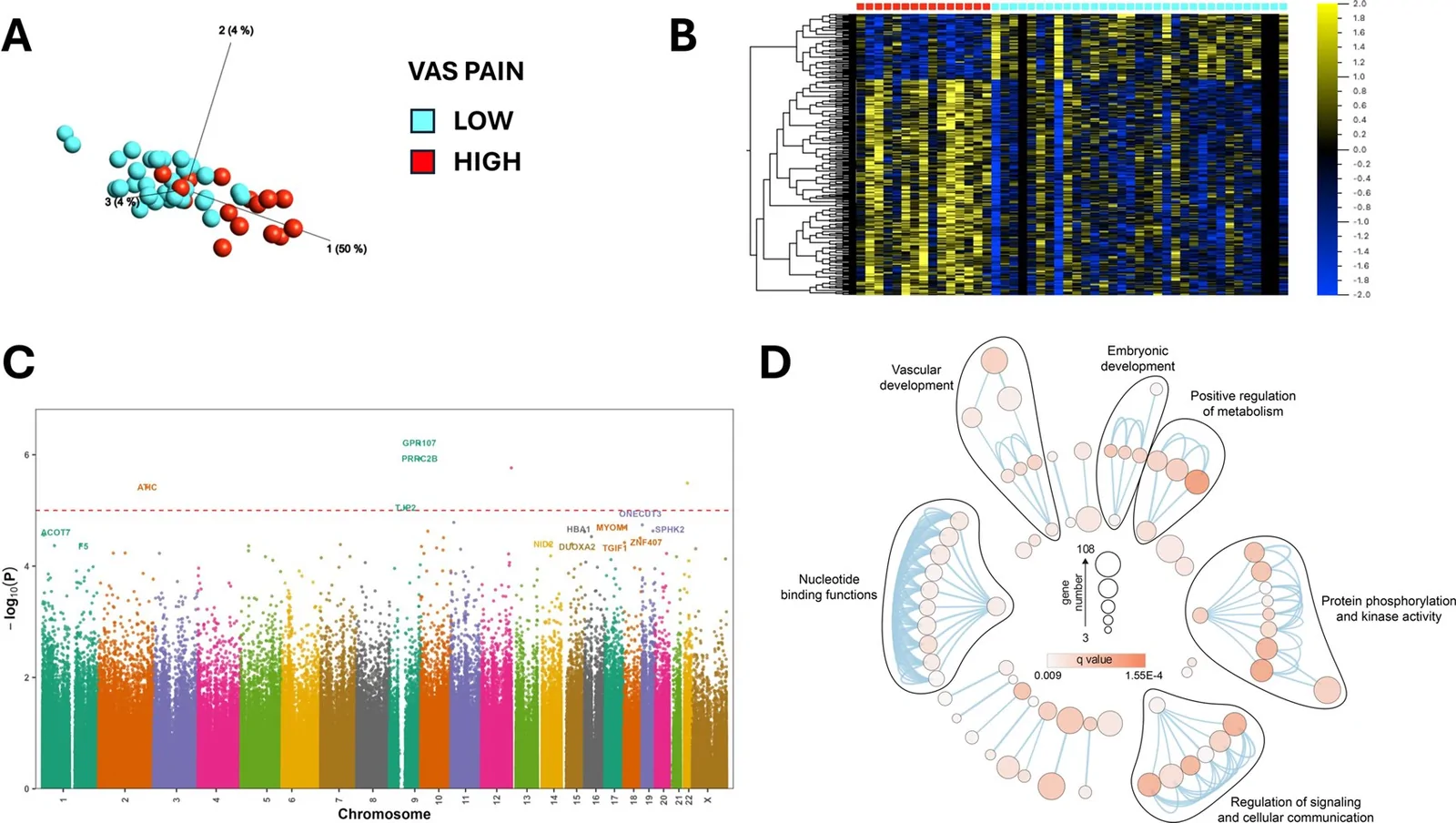
Genome-wide association for VAS for pain. (**A**) PCA of patients with low VAS for pain <35 (*n* = 33; light blue) and high VAS for pain ≥35 (*n* = 15; red). (**B**) Heatmap of CpG methylation patterns comparing patients with low VAS for pain (light blue) and high VAS for pain (red). (**C**) Manhattan plot showing the significance levels of each CpG locus with dichotomized VAS pain. Each dot represents a single significant CpG locus. Only the top genes of annotated CpGs are shown. (**D**) Pain associated pathways (low VAS for pain *vs* high VAS for pain). Enrichment map shows pathways significantly enriched (*P* < 0.01) for all significantly changed gene methylations identified. Nodes in the network represent pathways and similar pathways with many common genes are connected. Groups of similar pathways are indicated. Nodes are coloured according to *q*-value and the size of the nodes reflects the number of genes in the node as indicated in the figure. PCA: principal component analysis; VAS: visual analogue scale

We first dichotomized the cohort according to VAS for pain using a threshold of 35, as previously described for chronic musculoskeletal pain [17], to identify a group with ‘high pain’ (VAS for pain ≥35, *n* = 15) and one with ‘low pain’ (VAS for pain <35, *n* = 33). PCA revealed stratification for patients based on VAS for pain (Fig. 1A). No CpG sites passed the FDR threshold of 0.05, and therefore we applied a stringent raw *P*-value cutoff of 0.001 for analyses to minimize false positives. Using this threshold, the analysis identified 485 differentially methylated CpGs between participants with low and high pain (Table S2), belonging to 390 different genes, as visualized in the heatmap and in the Manhattan plot (Fig. 1B and C). The top 10 annotated CpGs associated with VAS for pain are detailed in Table 2. Several genes showed multiple CpGs linked to VAS for pain, including RAS P21 Protein Activator 3 (*RASA3*), with three CpGs associated with pain at *P* < 0.001, four CpGs at *P* < 0.01 and 17 at *P* < 0.05 (Table 3 and Table S3A). Gene set enrichment analysis (GSEA) was performed to identify clusters of functionally related pathways. The most impacted pathways were those related to nucleotide and ATP binding functions, protein phosphorylation and kinase activity, regulation of signalling and cellular communication, positive regulation of metabolism and biosynthesis, embryonic development, and vascular development (Table S4 and Fig. 1D). The gene sets most strongly correlated to pain were ‘positive regulation of metabolic process’ (GO: 0009893; *q* = 1.55 × 10^−4^), ‘macromolecule modification’ (GO: 0043412, *q* = 2.64 × 10^−4^), ‘regulation of signalling’ (GO: 0023051, *q* = 4.10 × 10^−4^) and ‘regulation of cell communication’ (GO: 0010646, q = 4.10 × 10^−4^) (Table S4). Alongside the dichotomized analyses, sensitivity analyses were performed by treating VAS pain score as continuous variables and applying linear models. After correcting for multiple testing, no CpG sites showed statistically significant results. The overlap between CpGs found in dichotomous and continuous analyses was 41%, as shown in Table S5 listing the relevant genes.

**Table 2:** Top annotated CpGs associated with high VAS for pain, fatigue and general health.

VAS for pain			VAS for fatigue			VAS for general health		
CpGs	Gene	*P*-value	CpGs	Gene	*P*-value	CpGs	Gene	*P*-value
cg00426626	*GPR107*	6.29 × 10^−7^	cg04700996	*GALNT9*	3.02 × 10^−6^	cg21873011	*ZNF214*	6.16 × 10^−7^
cg06539413	*PRRC2B*	1.17 × 10^−6^	cg23429735	*AP4E1*	6.95 × 10^−6^	cg14916917	*RPS23*	7.35 × 10^−7^
cg09837632	*ATIC*	3.83 × 10^−6^	cg17418779	*RGS5*	9.85 × 10^−6^	cg04775502	*C6orf114*	2.09 × 10^−6^
cg17195771	*TJP2*	8.97 × 10^−6^	cg08586426	*LMO2*	1.19 × 10^−5^	cg11987945	*LHFPL2*	2.83 × 10^−6^
cg08611625	*ONECUT3*	1.82 × 10^−5^	cg01468505	*ORC2L*	1.31 × 10^−5^	cg13196143	*GBA2*	5.02 × 10^−6^
cg06724776	*MYOM1*	2.03 × 10^−5^	cg21873011	*ZNF214*	1.65 × 10^−5^	cg15421037	*PRMT8*	8.53× 10^−6^
cg22480078	*SPHK2*	2.34 × 10^−5^	cg10794399	*AFF3*	1.66 × 10^−5^	cg16598111	*RASSF5*	9.60 × 10^−6^
cg08923355	*HBA1*	2.40 × 10^−5^	cg11904869	*PGAM1*	1.77 × 10^−5^	cg14080542	*C9orf93*	1.32 × 10^−5^
cg11078128	*ACOT7*	2.77 × 10^−5^	cg20370799	*PRUNE2*	2.12 × 10^−5^	cg26639820	*KRT78*	1.52 × 10^−5^
cg04356003	*ZNF407*	3.13 × 10^−5^	cg02561425	*COL6A3*	2.20 × 10^−5^	cg01330290	*MED12L*	1.54 × 10^−5^

VAS: visual analogue scale.

**Table 3:** Genes with multiple associated CpGs for VAS for pain, fatigue and health.

Pain			Fatigue			Health		
Gene	**Number of CpGs at *P*** **<** **0.001**	**Number of CpGs at *P*** **<** **0.01**	Gene	**Number of CpGs at *P*** **<** **0.001**	**Number of CpGs at *P*** **<** **0.01**	Gene	**Number of CpGs at *P*** **<** **0.001**	**Number of CpGs at *P*** **<** **0.01**
*RASA3*	3	4	*CAMTA1*	2	8	*SDK2*	2	12
*ATP11A*	2	9	*CUX1*	2	8	*MCF2L*	2	6
*LOC144571*	2	5	*GALNT9*	2	6	*CTBP1*	2	4
*RERE*	2	4	*GLRX2*	2	3	*SH3BP1*	2	4
*CDKN1A*	2	4	*DDX51*	2	3	*CUX1*	2	3
*PLEC1*	2	3	*AGAP1*	2	2	*KIFC3*	2	2
*PEX26*	2	2	*TSLP*	2	2	*TECPR2*	2	2
*CCDC64*	2	2				*RNF145*	2	2
*RUFY3*	2	2				*AMOTL2*	2	2
*C7orf63*	2	2						

List of genes with correlation to VAS for pain, fatigue and health at multiple CpG sites. Number of CpGs (two or more are listed) correlating at *P* = 0.05. VAS: visual analogue scale.

### Fatigue

**Figure 2:**
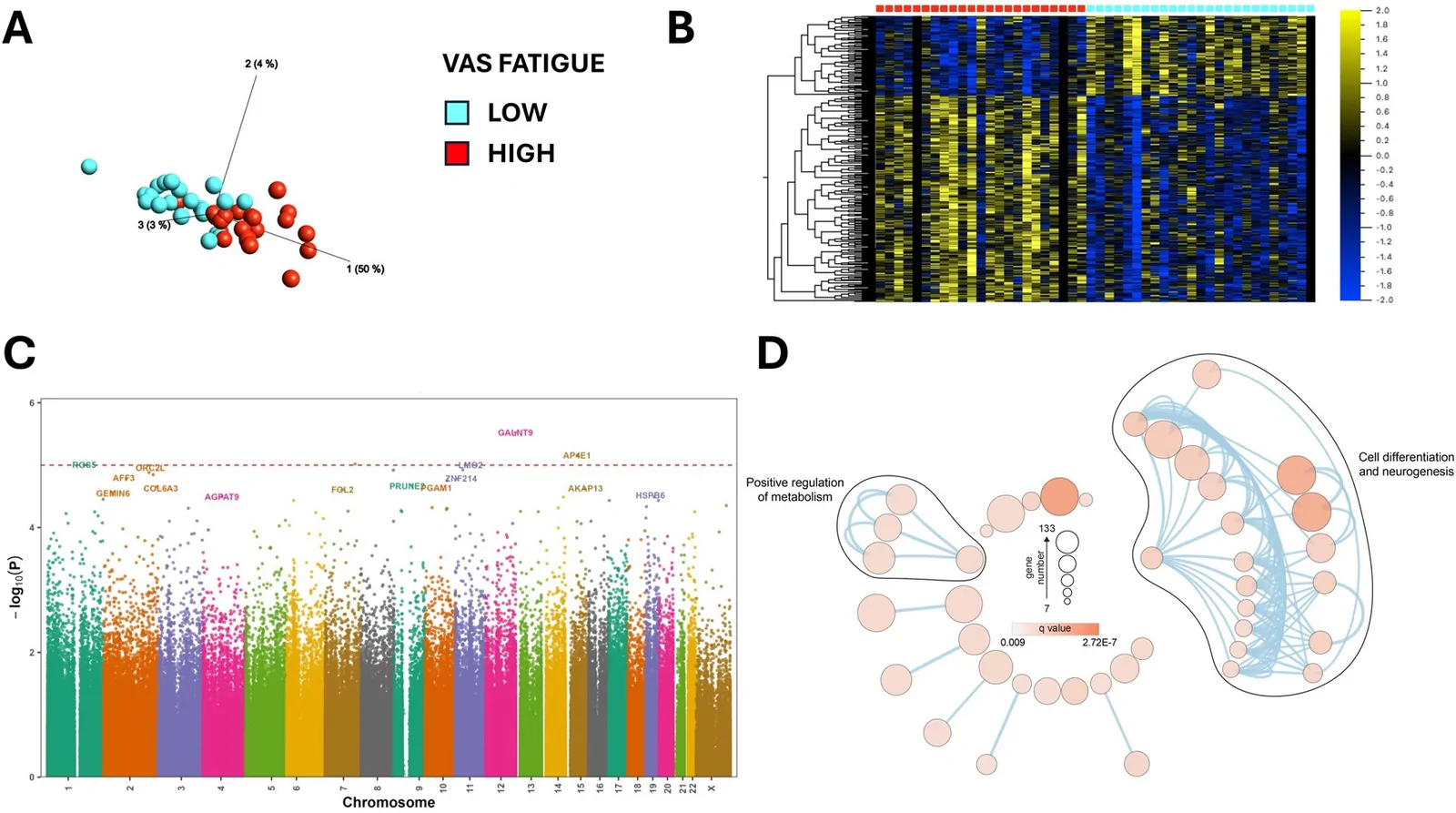
Genome-wide association for VAS for fatigue. (**A**) PCA of patients with low VAS for fatigue <35 (*n* = 25; light blue) and high VAS for fatigue ≥35 (*n* = 23 red). (**B**) Heatmap of CpG methylation patterns comparing patients with low VAS for fatigue (light blue) and high VAS for fatigue (red). (**C**) Manhattan plot showing the significance levels of each CpG locus with dichotomized VAS fatigue. Each dot represents a single significant CpG locus. Only the top genes of annotated CpGs are shown. (**D**) Fatigue associated pathways (low VAS for fatigue *vs* high VAS for fatigue). Enrichment map shows pathways significantly enriched (*P* < 0.01) for all significantly changed gene methylations identified. Nodes in the network represent pathways and similar pathways with many common genes are connected. Groups of similar pathways are indicated. Nodes are coloured according to *q*-value and the size of the nodes reflects the number of genes in the node as indicated in the figure. PCA: principal component analysis; VAS: visual analogue scale

PCA indicated stratification of patients based on VAS for fatigue, with 23 patients in the high fatigue group (VAS ≥ 35) and the remainder in the low fatigue group (Fig. 2A). Using the raw *P*-value cutoff of 0.001, the analysis revealed 591 differentially methylated CpGs between the low and high fatigue groups (Table S6), mapping to 479 different genes (Fig. 2B and C). The top 10 annotated CpGs associated with VAS for fatigue are listed in Table 2. Some genes showed multiple CpGs linked to VAS for fatigue, such as calmodulin binding transcription activator 1 (*CAMTA1*), with two CpGs associated with fatigue at *P* < 0.001, eight CpGs at *P* < 0.01 and 32 at *P* < 0.05 (Table 3 and Table S3B). For dichotomized VAS fatigue scores, GSEA identified two major clusters: positive regulation of metabolism, and cell differentiation and neurogenesis (Table S7 and Fig. 2D). The top enriched pathways were ‘nucleoplasm’ (GO: 0005654; *q* = 2.72 × 10^−7^), ‘cellular development processes’ (GO: 0048869; *q* = 2.05 × 10^−6^), ‘cell differentiation’ (GO: 0030154; *q* = 2.05 × 10^−6^) and ‘neurogenesis’ (GO: 0022008; *q* = 2.35 × 10^−6^) (Table S7). In addition to dichotomized analyses, sensitivity analyses were conducted by considering the VAS fatigue score as a continuous variable using linear models. After adjusting for multiple tests, no CpG sites reached statistical significance. Twenty percent of the CpGs identified overlapped between dichotomous and continuous approaches (Table S8).

### General health

**Figure 3:**
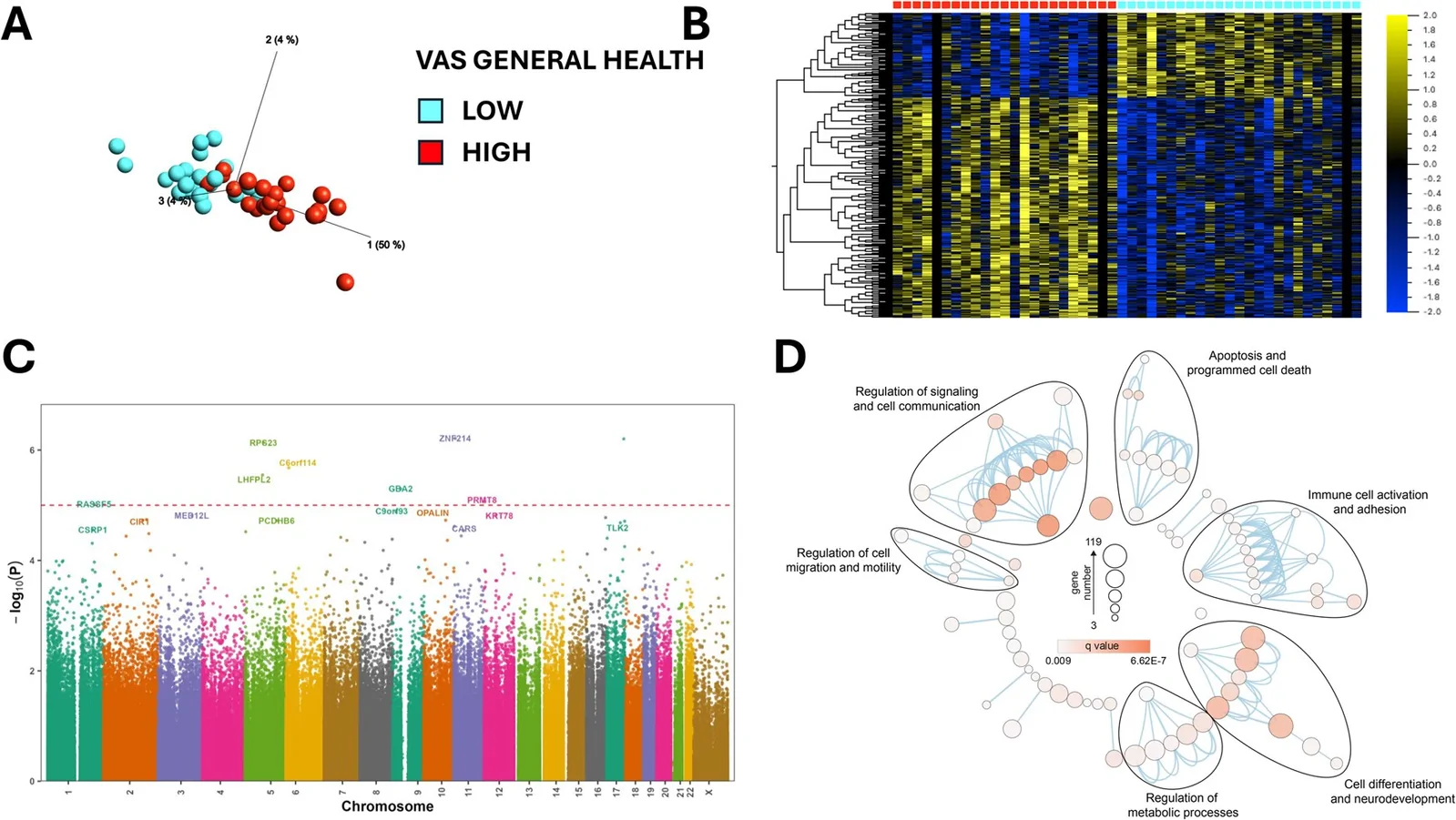
Genome-wide association for VAS for general health. (**A**) PCA of patients with low VAS for general health <35 (*n* = 25; light blue) and high VAS for general health ≥35 (*n* = 23; red). (**B**) Heatmap comparing CpG methylation between patients with low VAS for health (light blue) and high VAS for health (red). (**C**) Manhattan plot showing the significance levels of each CpG locus with VAS health as continuous variable. Each dot represents a single significant CpG locus. Only the top genes of annotated CpGs are shown. (**D**) Pathways associated with general health (low VAS for health *vs* high VAS for health). Enrichment map shows pathways significantly enriched (*P* < 0.01) for all significantly changed gene methylations identified. Nodes in the network represent pathways and similar pathways with many common genes are connected. Groups of similar pathways are indicated. Nodes are coloured according to *q*-value and the size of the nodes reflects the number of genes in the node as indicated in the figure. PCA: principal component analysis; VAS: visual analogue scale

PCA revealed clear stratification of patients based on VAS for general health, using a threshold of 35, with 23 patients in the group having VAS health ≥35 (Fig. 3A). By applying the raw *P*-value cutoff of 0.001, the analysis detected 577 differentially methylated CpGs between the low and high health scores (Table S9), belonging to 418 different genes. The results are detailed in the heatmap (Fig. 3B) and in the Manhattan plot (Fig. 3C). Table 2 outlines the top 10 CpGs associated with VAS for health. Some genes showed multiple CpGs linked to VAS for health, such as sidekick cell adhesion molecule 2 (*SDK2*), with two CpGs associated with health at *P* < 0.001, 12 CpGs at *P* < 0.01 and 23 at *P* < 0.05 (Table 3 and Table S3C). GSEA identified 81 pathways for dichotomized VAS health scores, which clustered into regulation of signalling and cell communication, immune cell activation and adhesion, cell differentiation and neurodevelopment regulation of cell migration and motility, apoptosis and programmed cell death, and regulation of metabolic processes (Fig. 3D). The pathway showing the strongest correlation was ‘regulation of signalling’ (GO: 0023051, *q* = 6.63 × 10^−7^), ‘regulation of signalling transduction’ (GO: 0009966, *q* = 1.00 × 10^−6^) and ‘regulation of cell communication’ (GO: 0010646, *q* = 1.00 × 10^−6^, Table S10). In addition to the dichotomized analyses, VAS scores for general health were analysed as continuous variables using linear models. No CpG sites reached statistical significance after multiple testing correction. Partial overlap was observed between CpGs identified in dichotomous and continuous analyses (46%, Table S11).

### Shared genes correlating with self-reported pain, fatigue and general health

Nine genes were identified with strong correlation to all three self-reported symptoms as shown in Fig. S2. These genes are enriched in key biological pathways including neurotrophic signalling and neuronal regulation, lipid and bile acid metabolism, RNA processing and tRNA modification, stress and transcriptional regulation, and cellular and structural elements (Table S12).

### Methylation of miRNA specific genes associated with VAS for pain, fatigue or general health

The methylation near or around the promoter of miRNA genes is known to regulate the expression of those genes [18], and therefore we specifically looked at the epigenome methylation of miRNA genes in our cohort. Four miRNA genes were associated with VAS for pain: MIR6850, MIR1268A, MIR548H3 and MIR3665 (Table S13A). Three more miRNA genes were associated with VAS for fatigue (MIR6764, MIR4535, MIR3665; Table S13B), whereas MIR1268A also correlated with VAS for health.

## Discussion

By performing an unbiased analysis of DNA methylation in blood from women with well-controlled, low-disease activity SLE with minimal chronic damage, we explored potential epigenetic differences associated with VAS scores for pain, fatigue and impaired general health. No CpG sites reached statistical significance after correction for multiple testing, suggesting that there are no robust epigenome-wide associations in this cohort. However, suggestive patterns of differential methylation were observed at nominal significance levels, indicating the possibility of subtle epigenetic differences related to these symptoms. Many of the CpGs showing these patterns mapped to genes or pathways previously implicated in neuronal signalling, development and modulation, raising the possibility that persistent pain and fatigue in well-controlled SLE may be associated with subtle, non-inflammatory neuronal epigenetic changes rather than solely residual inflammation. We have also identified several miRNA genes suggestively associated with self-reported pain, fatigue and general health.

To understand the correlation of reported scores for pain, fatigue and health with epigenetic changes in this well-characterized cohort of women with SLE, we have performed an unbiased analysis of DNA methylation patterns at CpG sites. The strongest association with VAS pain was observed for a CpG in *GPR107* gene, which encodes a receptor for neuronostatin [19]. Neuronostatin has multiple functions including antinociceptive activity [20], which is dependent on GPR107 [21]. Another key gene, *SPHK2*, encodes a sphingosine kinase isozyme that converts sphingosine into sphingosine 1-phosphate, a key component in central sensitization and pain signalling [22]. *SPHK2* was also strongly associated with VAS fatigue and health, highlighting its broader relevance in these symptoms in SLE. A CpG site in the haemoglobin subunit α 1 (*HBA1*) gene showed strong association with VAS pain. HBA1 is a precursor of haemopressin [23], a bioactive peptide that modulates cannabinoid receptor type 1 in the central nervous system, influencing pain perception and neuromodulation. Taken together, these findings highlight the potential role of neuromodulation in pain perception in SLE.

Some genes showed multiple CpG sites associated with pain, with *RASA3* gene ranking highest based on the number of CpGs correlated with pain at the predefined significance threshold. RASA3 is involved in T cell trafficking [24]; in a recent study DNA methylation was decreased in *RASA3* after flare remission in patients with SLE [25]. The location of the identified differentially methylated CpGs within the gene body of *RASA3* suggests a more complex mode of regulation, as opposed to the suppressive regulation typically associated with promoter methylation. Interestingly, another pain-related gene with multiple CpG sites was *RERE*, which encodes a widely expressed nuclear receptor co-regulator with neurodevelopmental function, regulating retinoid signalling during development [26]. Notably, *RERE* was located within a previously reported SLE susceptibility locus [27], suggesting a possible link between genetic susceptibility and epigenetic variation at this gene. RERE is expressed in lymphoid cells [28] and is genetically associated with generalized vitiligo [29].

Pathway analysis showed that high VAS pain scores were associated with metabolism, cell signalling and developmental processes. Notably, several of the genes associated with pain were involved in pain perception and neuromodulation in the central nervous system, suggesting that both metabolic and neuroimmune mechanisms may contribute to pain in SLE. Previous studies have shown that chronic pain sensitization is characterized by epigenetic reprogramming, indicating its possible role in the persistence of this symptom [30, 31]. DNA methylation changes observed in animal models of neuropathic pain, persisting long after injury, reinforce the role of methylation in maintaining chronic pain [32]. The expression of DNA methyltransferases is pain-type-specific, with DNA methyltransferases 3a generally upregulated in neuropathic and inflammatory pain and DNA methyltransferases 3 b downregulation facilitating gene hypomethylation in chronic pain [33–35]. The involvement of DNA methylation in chronic pain is further underscored by the actions of epigenetic readers like methyl CpG-binding protein 2, which becomes phosphorylated and inactivated in inflammatory pain models, and methyl-CpG binding domain protein 1, which has been linked to hypersensitivity in neuropathic pain when deficient [36, 37].

None of the top genes found associated with VAS fatigue have previously been linked to fatigue, which highlights the potential for novel pathways underlying this symptom in SLE. However, some genes have previously been associated with SLE pathophysiology and activity. For example, regulator of G protein signalling (*RGS5*) has been suggested as a marker of vascular dysfunction in SLE [38], and its protein may contribute to neuroinflammation via tumour necrosis factor signalling [39], potentially linking vascular and neural mechanisms to fatigue. Polymorphisms in AF4/FMR2 family, member 3 (*AFF3*) have been previously associated with risk for autoimmune conditions, including SLE [40], but also to neurodevelopmental phenotypes such as intellectual disability [41], raising the possibility that AFF3-mediated transcriptional regulation could influence both immune and neural pathways relevant to fatigue. Finally, *COL6A3* encodes for collagen α-3(VI) chain, and is a biomarker of lupus nephritis [42]. Both *CAMTA1* and cut like homeobox 1 (*CUX1*) genes showed multiple CpGs associated with VAS fatigue. *CAMTA1*, a calmodulin-binding transcription factor, is highly expressed in the brain and regulates neuronal development, differentiation, survival and long-term memory [43]. CAMTA1 shows reduced RNA expression in T cells from patients with SLE and correlates with disease activity, suggesting that CAMTA1 may influence signalling pathways in both immune and neural cells, potentially linking immunological and neurodevelopmental processes to fatigue. *CUX1* has been recently suggested to play a pivotal role in SLE progression [38]. Together, these findings suggest that fatigue in SLE may involve interacting immune, vascular and central nervous system pathways influenced by genes regulating neuroinflammation, neuronal function and transcription.

Pathway analysis showed that genes associated with reported fatigue did not cluster in inflammation or immune response pathways but primarily converged on neurological pathways, specifically those related to neuronal development and synaptic signalling. These findings may offer a possible explanation as to why symptoms of chronic fatigue and pain persist in patients with otherwise well-controlled disease and do not classically respond to conventional immunosuppressive therapy. Neuroplasticity is the ability of the nervous system to adapt to external stimuli and plays an important role in the transition from acute to chronic pain and in the long-term maintenance of pain [44]. The role of neuroplasticity in fatigue is less well studied. It has been previously hypothesized that the persistent inflammatory state in SLE with recurring flare-ups may affect central nervous system processes involved in fatigue and pain [45]. Our results highlight the possible role of altered epigenetic regulation in maintaining pain and fatigue in SLE patients through altered neurological pathways, even when the disease activity is low. If confirmed, these findings could pave the way for therapeutic approaches targeting neuroplasticity and neuronal signalling rather than solely modulating immune activity, potentially improving pain and fatigue outcomes in SLE. Moreover, understanding the epigenetic basis of persistent pain and fatigue in well-controlled SLE may lead to the discovery of biomarkers able to identify patients at higher risk for these symptoms, allowing earlier and personalized interventions.

Evidence of the involvement of neuronal modulation in the perception of pain, fatigue and poor health in well-controlled patients with SLE is also supported by the observation that several of the nine shared genes associated with all three symptoms are functionally linked to the nervous system, rather than inflammatory or immune pathways. The strongest association for all three symptoms was for *BDNF*, which plays pivotal roles in neuronal survival and growth and synaptic plasticity. We have also recently reported that epigenetic regulation of *BDNF* is associated with SLE disease activity [46]. Another shared gene involved in the perception of pain, fatigue and health was TGFB induced factor homeobox 1 (*TGIF1*), whose protein is involved in normal brain development. Interestingly, a recent study has shown that autoantibody against TGIF1 were associated with SLE and highly represented in patients with central nervous system involvement [47], further supporting a potential link between neurodevelopmental pathways, autoimmunity and symptom burden in SLE.

DNA methylation is considered relatively stable compared with gene expression, which can fluctuate in response to transient factors such as inflammation, medications, or circadian variation. In this study, participants had low disease activity, so the observed associations between DNA methylation and patient-reported symptoms likely reflect more long-lasting biological changes rather than short-term fluctuations. This suggests that persistent pain, fatigue and overall poor health in SLE may be linked to long-term epigenetic regulation of specific pathways, particularly those involved in neuronal function, rather than being solely driven by transient inflammation during flares. Several miRNA genes showed differential methylation in relation to pain, fatigue and impaired health. Altered promoter methylation of MIR6850 in patients with higher pain, and of MIR4535 in patients with higher fatigue, may contribute to dysregulation of these miRNAs. MIR6850 has previously been linked to centrally mediated abdominal pain syndrome [48], while MIR4535 was reported to be downregulated in cubital tunnel syndrome, the second most common peripheral nerve compression disorder [49]. Together, these findings suggest that epigenetic dysregulation of specific miRNAs may underlie previously reported expression changes and may play a role in neuropathic mechanisms and the regulation of pain perception.

One of the primary limitations of this study is the relatively small cohort size. Furthermore, a key limitation is that no CpG sites reached statistical significance after correction for multiple testing. Given the large number of loci examined, the use of a nominal threshold (*P* < 0.001) increases the risk that some of the observed associations may reflect random variation. Therefore, the individual CpG findings should be interpreted with caution. The results are best viewed as exploratory and hypothesis-generating. Future studies with larger sample sizes are required to validate these findings and determine their robustness. Another limitation is that our cohort included only women, which limits the applicability of our conclusions to men. Sex-related differences in epigenetic mechanisms as well as in the perception of pain and fatigue are well-known [50], and the exclusion of male participants means that our findings may not be generalizable to men. Furthermore, as our study focused on DNA methylation, it did not explore other epigenetic changes such as histone modifications or non-coding RNA interactions, which may also play a role in processes associated with SLE.

This study indicates that DNA methylation influences reported pain, fatigue and general health in women with well-controlled SLE. Our findings show limited involvement of inflammatory pathways but suggest that epigenetic changes in genes involved in neuronal development and synaptic signalling could be involved in the chronicization of pain and fatigue in patients with SLE, even when the disease activity is low. By uncovering targets and pathways that potentially sustain these symptoms, these results could contribute to the future development of new strategies to manage pain and fatigue in SLE, including epigenetically focused treatments. Further research is needed to understand the specific pathways and mechanisms through which epigenetic modifications affect the self-perception of pain, fatigue and health in patients with SLE.

## Supplementary Material

keag392_Supplementary_Data

## Data Availability

The data underlying this article will be shared on reasonable request to the corresponding author.
